# Adherence with reporting of ethical standards in COVID-19 human studies: a rapid review

**DOI:** 10.1186/s12910-021-00649-9

**Published:** 2021-06-28

**Authors:** Lydia O’Sullivan, Ronan P. Killeen, Peter Doran, Rachel K. Crowley

**Affiliations:** 1 School of Medicine, University College Dublin, Dublin 4, D04 V1W8 Ireland; 2 Ethics and Medical Research Committee, Saint Vincent’s University Hospital, Dublin 4, D04 T6F4 Ireland; 3 Saint Vincent’s University Hospital, Dublin 4, D04 T6F4 Ireland; 4 Health Research Board-Trials Methodology Research Network, National University of Ireland, Galway, H91 TK33 Aras Moyola Ireland

**Keywords:** Clinical research, Clinical trials, Good Clinical Practice, Research ethics, Research Ethics Committee, Pandemic, COVID-19, Patient and Public Involvement, Declaration of Helsinki, Informed Consent

## Abstract

**Background:**

Patients with COVID-19 may feel under pressure to participate in research during the pandemic. Safeguards to protect research participants include ethical guidelines [e.g. Declaration of Helsinki and good clinical practice (GCP)], legislation to protect participants’ privacy, research ethics committees (RECs) and informed consent. The International Committee of Medical Journal Editors (ICMJE) advises researchers to document compliance with these safeguards. Adherence to publication guidelines has been suboptimal in other specialty fields. The aim of this rapid review was to determine whether COVID-19 human research publications report compliance with these ethical safeguards.

**Methods:**

A rapid systematic literature review was conducted in MEDLINE using the search term ‘COVID-19’. The search was performed in April 2020 with no start date and repeated to include articles published in November 2020. Filters were ‘Full free text available’ and ‘English Language’. Two reviewers assessed article title, abstracts and full texts. Non-COVID-19 articles and non-clinical studies were excluded. Independent reviewers conducted a second assessment of a random 20% of articles. The outcomes included reporting of compliance with the Declaration of Helsinki and GCP, REC approval, informed consent and participant privacy.

**Results:**

The searches yielded 1275 and 1942 articles of which 247 and 717 were deemed eligible, from the April  search and November respectively. The majority of journals had editorial policies which purported to comply with ICMJE ethical standards. Reporting of compliance with ethical guidelines was low across all study types but was higher in the November search for case series and observational studies. Reporting of informed consent for case studies and observational studies was higher in the November search, but similar for case series. Overall, participant confidentiality was maintained but some case studies included a combination of details which would have enabled participant identification. Reporting of REC approval was higher in the November search for observational studies.

**Conclusions:**

While the majority of journal’s editorial policies purported to support the ethical safeguards, many COVID-19 clinical research publications identified in this rapid review lacked documentation of these important safeguards for research participants. In order to promote public trust, ethical declarations should be included consistently.

**Supplementary Information:**

The online version contains supplementary material available at 10.1186/s12910-021-00649-9.

## Background

The global pandemic spread of a coronavirus has led to the respiratory disease named COVID-19 [1]. COVID-19 research is being published at an unprecedented rate but questions have been asked about the methodological quality of this research [2–5]. During a pandemic, research participants may be more vulnerable because of reduced contact with family or physicians to discuss research participation, and they may agree to investigational treatments because of health anxiety or confusion of research with clinical practice.

One of the safeguards to protect participants in research is good clinical practice (GCP) training and certification for investigators and compliance with these standards [6]. Additional protections for participants include legislation which protects participants’ privacy and autonomy, review of the proposed research by research ethics committees (RECs) and ethical publishing standards of medical journals. Two further safeguards not dealt with in this review paper include regulatory authority inspections and monitoring of ethical conduct by trial sponsors.

GCP guidelines, which are consistent with the Declaration of Helsinki [7], were prepared for Clinical Trials of Investigational Medicinal Products (CTIMPs), however, the ethical standards contained therein can be applied to all research involving human participants. GCP states that ‘compliance with this standard provides public assurance that the rights, safety and well-being of trial subjects are protected’[6]. Legislation, such as the European Union’s (EU) General Data Protection Regulation (GDPR) of 2018 provides legal rights to members of the public with respect to their personal data [8] and these rights protect the privacy of research participants. RECs are responsible for ensuring the safety of research participants and among other tasks, review whether there is an ethical process in place for participants to provide informed consent. The International Committee of Medical Journal Editors (ICMJE) recommendations state that publications should specify if experimental research on human participants has been conducted in line with the Declaration of Helsinki and the relevant REC, whether informed consent was provided and emphasises that participants’ privacy should be protected [9]. Clinical research activity is also subject to the laws of the country where it is conducted, and if researchers provide the name of the REC in publications, this allows readers to locate the relevant REC and assess their operating standards of ethical review, either by consulting their website or contacting a representative. Similarly, the Council of Science Editors stated that ‘Editors can and should play their part in upholding ethical standards by refusing to publish reports of work that violates human rights even if the work seems scientifically valid and important’ [10]. The Committee on Publication Ethics (COPE) provides specific guidance on the publication of case studies, including making efforts to ensure that investigators have obtained written informed consent from the participant [11]. In this way, publishers of medical journals can exert indirect pressure on investigators to conduct ethically sound research [12]. Many medical and biomedical journals refer to compliance with the ICMJE and COPE guidelines or advise authors that they should comply with the ethical safeguards described above within their editorial policies.

In a time of pandemic, the effort made by clinicians to conduct research is to be lauded [13]. It is not the intent of these authors to criticize the research effort made by clinicians, sometimes under challenging conditions. However, it is also imperative to maintain public confidence in research by conducting good quality studies and by reporting the ethical safeguards in place for the study participants. Medical journals also have an important role to play in gatekeeping ethical standards, and thus maintaining public trust in human research. The publication of papers in *The Lancet* and the *New England Journal of Medicine* followed rapidly by Expressions of Concern and then retraction, has been widely discussed in the mainstream media and this coverage highlights the importance of conducting good quality research in which the public can have confidence [14, 15]. We acknowledge that reporting ethical standards in academic literature is not the only vehicle to promote trust in researchers. However, we propose that public trust will be undermined if researchers do not document their compliance with ethical safeguards at every opportunity. Among other things, this includes reporting REC approval and emphasising patient autonomy e.g. in Participant Information Leaflets and involving the public in co-designing research. Since reporting of the results of clinical research often takes place in the first instance via medical journals, followed by dissemination into the media, we feel that it is important to document compliance in this way. In a time of fake news and misinformation, it is important that researchers are clear and transparent about their research practices.

These various systems (ethical guidelines, legislation, REC review, informed consent and publication standards) function synergistically to ensure research is safe, promoting public trust. It is important to note that case studies and case series are generally not considered research as they are simply reports of observations and reflections upon the learning points or management options. For this reason, many institutions ask that patients provide their consent for publication, but do not require REC review. Similarly, as the Declaration of Helsinki and GCP apply to the research context, it wouldn’t be considered necessary to specifically declare compliance with these standards in publications. However, the individuals described in case studies and case series have the same rights to professional treatment and privacy as any other patient or research participant. It has become increasingly clear that the public want transparency with regard to decision-making [16] and are becoming more aware of trial design and conduct [17]. It is therefore critical that researchers are clear in publications about the kinds of ethical safeguards which they use or why they were not applicable.

The aim of this rapid review was to assess how well journal articles of COVID-19 research, included in MEDLINE, report ethical declarations advised by the ICMJE such as protection of participants’ privacy, compliance with the Declaration of Helsinki or GCP, REC approval and informed consent. Two searches were conducted: one at the beginning of the pandemic (January to April 2020) and a second one when COVID-19 research had become more established (November 2020).

## Methods

The protocol for this rapid review was published on the F1000 Health Research Board Open Research platform [18]. Due to the rapidly changing research environment during the COVID-19 pandemic, a decision was made to repeat the first search (completed in April 2020), in November 2020 so that comparisons could be drawn between the two time periods. The review was conducted in accordance with the Preferred Reporting Items for Systematic Reviews and Meta-Analysis (PRISMA) [19]—see Additional file 1: PRISMA checklist.

### Search strategy

The search for this rapid review is described with reference to the PRISMA-S checklist [20]—see Additional file 2. The aim of this review was to gain a broadly representative sample of the ethical declarations in published COVID-19 medical research. Therefore, for this rapid review, PubMed (MEDLINE), the largest, freely-available, medical database was selected. For the same reason, reference lists were not examined. The term ‘COVID-19’ was searched for within Titles and Abstracts, with filters for ‘Full free text’ and ‘English language’. While restricting the search to ‘Full free text’ is a limitation of this review, more than 50 publishers have made COVID-19 research publications open access through PubMed via the COVID-19 Initiative [21]. This enabled the authors to easily gain a broadly representative sample of COVID-19 articles. The first search took place on 14^th^ April 2020 and included all articles published up until this point. The second search took place on 18^th^ Dec 2020 and included all articles published in November 2020. In order to have a similar-sized group of articles for comparison with the April 2020 search, a random 50% of the articles from the November 2020 search were selected for review using the random sampling function in Microsoft Excel.

### Study selection

Two reviewers assessed the article titles, abstracts and full texts and classified them into:

- Case Studies
- Case Series
- Observational Studies
- Clinical Trials of Investigational Medicinal Products (CTIMPs)
- Studies which were not CTIMPs

Articles were excluded if they were:

- Reviews, Systematic Reviews or protocols for either
- Non-clinical studies (e.g., animals studies, laboratory studies with no human data, epidemiological or modelling studies)
- Research not relating to COVID-19
- Protocols for clinical trials
- Commentaries, Corrigenda, Letters to the Editor (including editorials or society position statements for guidelines)

Two independent reviewers conducted a second assessment of a random 20% of articles from the April 2020 search; one independent reviewer conducted a second assessment of a random 20% of articles from the November 2020 search. The articles for the second assessment were selected using the random sampling function in Microsoft Excel. Authors of individual papers were not contacted to request the full text of inaccessible papers, due to time constraints.

### Data extraction and analysis

Data extraction was completed in Excel by two authors using a *proforma* spreadsheet—see Additional file 3. The outcomes included, among others, the ethical standards adopted by Yank and Rennie [22]: the reporting of compliance with the Declaration of Helsinki or GCP, an assessment regarding whether participants’ privacy had been maintained, approval of the research by a REC and informed consent from participants. These ethical standards also reflect the recommendations of the ICMJE. Since REC approval is not generally required for case studies or series, REC approval was only reported for observational studies, CTIMPs and other non-CTIMP studies. If a consent waiver was granted, this was also reported, along with the reason, if provided by the publication. The editorial policy of each journal identified in the rapid review was also examined to determine what proportion of the journals purported to comply with the ICMJE and COPE guidelines and four key ethical parameters (compliance with the Declaration of Helsinki or GCP, participant privacy, REC review and informed consent). A chi-squared test was performed to compare the April and November searches. A p-value of 0.05 was considered significant.

### Risk of bias and critical appraisal

The aim of this rapid systematic review was to assess the reporting of ethical declarations, rather than quantitatively assess healthcare interventions or to assess the methodological quality of clinical trials. Therefore, neither risk of bias or critical appraisal were applicable.

### Patient and public involvement

Patients and the public were not involved in the design and conduct of this study. However, two members of the public who had been patients and relatives of patients at this institution volunteered to assist with research and were asked if they would like to review a manuscript regarding publication ethics. Neither of these contributors had a medical or research background. They were given an early draft of the paper and were asked to review it and provide feedback to the authors, which was incorporated into the paper. They were not involved in the design of the research question or review of the literature, nor of the discussion points, therefore they are acknowledged in the paper but not included for authorship. They both gave their permission.

## Results

### Article/study inclusion

**Fig. 1:**
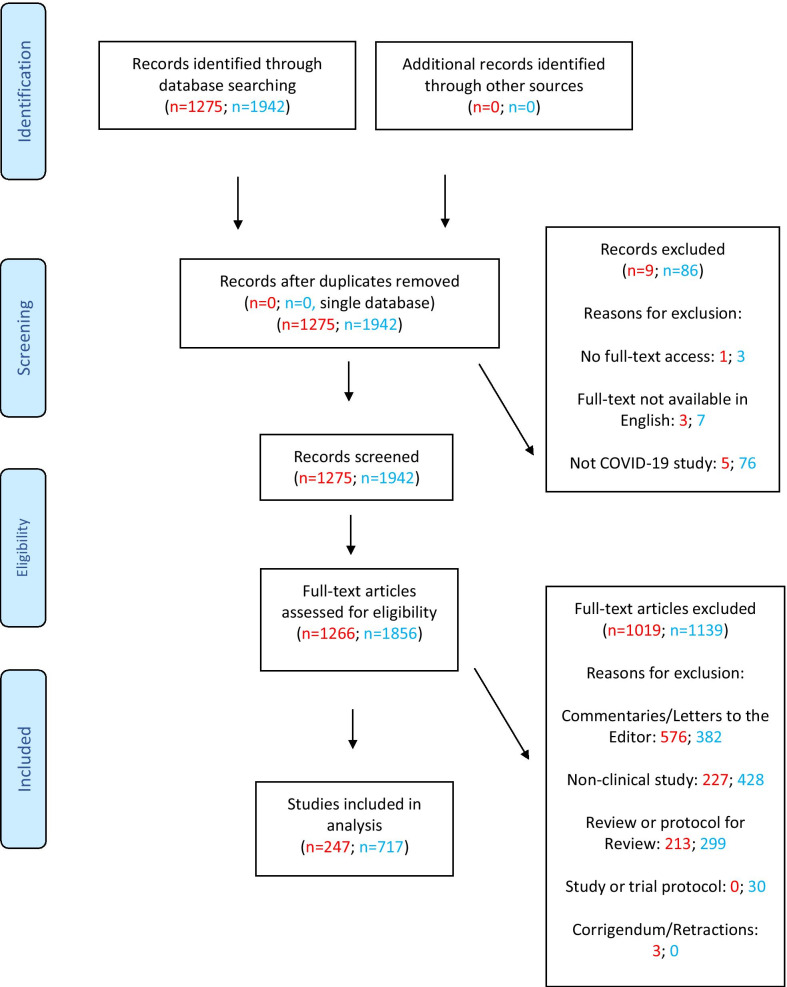
PRISMA Flowchart describing the results for both the April and November searches

The April 2020 search yielded 1275 articles, of which 247 were deemed eligible for the analysis. The November 2020 search produced 3877 articles of which a random 50% were selected (1942 articles)—of these, 717 articles were deemed eligible for the analysis. The PRISMA flowchart provides details of both searches—see Fig. 1, with the April 2020 search in red and the November 2020 search in blue. Discordance between independent assessors was less than 20%; this was resolved by assessor discussion to yield the list of final studies.

### Characteristics of included studies

Included articles were publications of case studies: 68 and 71; case series: 120 and 48; observational studies: 55 and 385; CTIMPs: 2 and 3; studies (non-CTIMPs): 0 and 18, for the April and November 2020 searches respectively. Summary data for all included studies are included Additional files 4 and 5.

### Editorial policies

Table 1 outlines whether the editorial policies reported by journals where the included studies were published included compliance with the ICMJE, COPE or similar ethical standards. 68.4% and 69.3% of journals identified in this rapid review purported to support the ICMJE guidelines in the April and November searches respectively. 26.3% and 23% claimed to support the COPE guidelines or specifically mentioned the key four ethical parameters (compliance with the Declaration of Helsinki or GCP, participant privacy, REC review and informed consent).

**Table 1:** Editorial policies reported by journals of included studies

	April search n = 247 (%)	Nov search n = 717 (%)
ICMJE	68.4	69.3
COPE or statement of 4 key ethical principles in editorial policy*	26.3	23
No statement of compliance with any guidelines or ethical principles	2.1	5.9
Not applicable: non-journal publications (e.g. bulletin)	3.2	1.8

*ICMJE* International Committee of Medical Journal Editors, *COPE* Committee on Publication Ethics

*Compliance with the Declaration of Helsinki or GCP, participant privacy, REC review and informed consent

### Compliance with ethical guidelines: Declaration of Helsinki or GCP

There were similar, low levels of reporting of compliance with the Declaration of Helsinki or GCP at both timepoints for case studies: 4.4% and 4% for April and November respectively. However, there were higher levels of compliance in the November search for case series: 20% compared to 1.7% and for observational studies: 18% compared to 12.7%. However, as noted previously, since the Declaration of Helsinki and GCP specifically apply to research and case studies/series are not considered research by most practitioners, it wouldn’t be considered necessary to specifically declare compliance with these standards in publications.

### Informed consent

Table 2 outlines whether included publications reported if informed consent was documented in the publications. Publications with no documentation of informed consent decreased for both case studies and observational studies: 70.6% (April) and 38% (November); 92% (April) to 36% (November) respectively. The rates were similar for case series: 71.1% in April compared to 75% in November.

**Table 2:** Summary of statements regarding consent and compliance with ethical guidelines

	Case study n = (%)		Case series n = (%)		Observational study n = (%)		CTIMPs n = (%)		Studies/trials (non-CTIMPs) n = (%)	
	April search n = 68	Nov search n = 71	April search n = 120	Nov search n = 240	April search n = 55	Nov search n = 385	April search n = 2	Nov search n = 3	April search n = 0	Nov search n = 18
No consent specified in article	48 (70.6%)*	27 (38%)*	86 (71.7%)	179 (75%)	23 (92%)	139 (36%)	2 (50%)	0 (0%)	-	2 (11%)
Written consent	11 (16.2%)	14 (20%)	17 (14.2%)	32 (13%)	14 (25.5%)	80 (21%)	2 (50%)	3 (100%)	–	8 (45%)
Article does not specify if verbal or written	6 (8.8%)	17 (24%)	10 (8.3%)	17 (7%)	3 (5.5%)	41 (11%)	0 (0%)	0 (0%)	–	6 (33%)
Verbal consent	2 (2.9%)	1 (1%)	2 (1.6%)	7 (3%)	2 (3.6%)	32 (8%)	0 (0%)	0 (0%)	–	2 (11%)
Electronic	0 (0%)	0 (0%)	0 (0%)	2 (0.8%)	3 (5.5%)	57 (15%)	0 (0%)	0 (0%)	–	0 (0%)
Not applicable—RIP	1 (1.5%)	11 (16%)	4 (3.3%)	3 (1.2%)	0 (0%)	0 (0%)	N/A	0 (0%)	–	0 (0%)
Assent	0 (0%)	1 (1%)	1 (0.8%)	0 (0%)	0 (0%)	0 (0%)	0 (0%)	0 (0%)	–	0 (0%)
Consent implied (completion of a survey)	–	–	–	–	0 (0%)	36 (9%)	–	–	–	–
Consent waived	3 (4.4%)	0 (0%)	25 (20.8%)	67 (28%)	10 (18.2%)	0 (0%)	–	–	–	–
Waived by Unknown	2 (2.9%)	0 (0%)	1 (0.8%)	9 (13%)	2 (3.6%)	–	–	–	–	–
Waived by REC	1 (1.5%)	0 (0%)	24 (20%)	54 (81%)	0 (0%)	–	–	–	–	–
Waived by other (local committee or law)	0 (0%)	0 (0%)	0 (0%)	4 (6%)	2 (3.6%)	0 (0%)	0 (0%)	0 (0%)	–	0 (0%)
Wording of consent, if provided	n = 11	n = 31	n = 17	n = 57	n = 14	n = 234	n = 2	n = 3	n = 0	n = 16
‘Written Informed Consent’/Strict definition ***	5 (45.5%)	13 (42%)	13 (76.5%)**	28 (49%)**	6 (43%)	123 (53%)	2 (50%)	3 (100%)	–	5 (31%)
Journal editor has copy of written consent form	0 (0%)	6 (8.5%)	0 (0%)	0 (0%)	–	–	–	–	–	–

*CTIMP* Clinical Trial of Investigational Medicinal Products, *RIP* participants(s) had died; *REC* Research Ethics Committee, *Nov* November

^*^p < 0.01; **p < 0.05 [chi-squared test]; *** ‘Strict definition’ of consent, as described by Yank and Rennie[22]

### Patient confidentiality

No patient names were published in articles identified in either search but other identifiers were described in the case studies and case series. For case studies, there was a decrease in the combination of age, gender and travel history being reported from 37% in the April search to 4% in the November search. There was a similar decrease in the combination of age, gender, travel history and occupation reported in case studies from 13.2 to 1.4%. However, the authors also noted some detailed descriptions of places of work, occupation and travel that, in the opinion of the authors and under EU GDPR, would allow participants or their acquaintances to identify the individuals described [23–29]. In the case series, aggregate-only data were published in 45% and 76% of the April and November publications respectively, which provided protection against identification of participants. However, in one case series from the November search, the initials of participants were included [30].

### REC approval

Table 3 summarises the reporting of REC review in the included articles. An increase in REC approval was reported in the November search for observational studies—80%, compared to 70.1%. A similar proportion of RECs were named in publications of observational studies from both searches 67% (April) and 65% (November) but a higher proportion of these RECs could be found via an online search: 77% (November); 43% (April).

**Table 3:** Summary of Research Ethics Committee for observational studies and clinical trials

	Observational studies n = (%)		CTIMPs n = (%)		Studies (non-CTIMPs) n = (%)	
	April search n = 55	Nov search n = 385	April search n = 4	Nov search n = 3	April search n = 0	Nov search n = 18
REC approval specified in article	39 (70.1%)	306 (80%)	4 (100%)	3 (100%)	0 (0%)	16 (89%)
If has REC approval and REC is named, can it be found online?	n = 37	n = 251	n = 2	n = 3	n = 0	n = 13
Yes	16 (43%)*	194 (77%)*	1 (50%)	1 (33%)	0 (0%)	9 (69%)

*REC* Research Ethics Committee, *CTIMP* Clinical Trial of Investigational Medicinal Products, *Nov* November

^*^p < 0.01 [chi-squared test]

### CTIMPs and non-CTIMP studies

Due to the very small number of CTIMPs and non-CTIMP studies identified in this review, it is not possible to draw any meaningful conclusions from these results. All of the CTIMPs had been registered and documented REC approval.

## Discussion

### Summary of key findings

Reporting of compliance with the Declaration of Helsinki and GCP was low across all study types and in both searches. Documentation of Informed Consent in publications of case studies and observational studies improved when the April and November searches were compared, although the rates for case series were comparable. Similarly, rates of reporting of REC approval for observational studies increased. There was also a decrease in publication of a combination of potentially identifying features in case studies between the April and November searches. Nonetheless, it is not clear why ethical safeguards are not reported more consistently in COVID-19 human studies. In addition, some publications reported descriptions of participants which would enable their recognition.

### Patient Confidentiality

Preservation of patient confidentiality is a core clinical value and publication of data that can be identified is undesirable. The ICMJE guidelines state that ‘Patients have a right to privacy that should not be violated without informed consent’ [9]. It is clear from online COVID-19 fora (e.g.www.flutrackers.com), the REC waivers reported in this rapid review and the examples provided of potentially identifying information, that different levels of patient confidentiality are accepted in different jurisdictions. Yoshida’s survey of patient consent to publish medical information in 491 medical journals similarly showed considerable variation, and found only 40% of journals required consent [31]. Under GDPR legislation in the EU, more data than name alone are considered identifiers and the Irish interpretation of this legislation is that formal consent for publication of personal data is required [8, 32]. It has been argued that releasing information about individuals diagnosed with COVID-19 is in the public interest. In Ireland the public release of information about individual cases of COVID-19 was limited by the Irish Government, in order to maintain trust in the public health services and to encourage a more complete contact declaration for tracing [33]. When case studies or series are reported in medical journals, a balance must be sought in publishing pertinent aspects of the patient’s medical history, and acknowledging the patient’s right to privacy [34].

### REC approval and informed consent

The ICMJE guidelines state that researchers should obtain approval from a REC and ‘When informed consent has been obtained, it should be indicated in the published article’ [9]. This rapid review showed that the publication requirements of journals did not result in reports of consent or REC review in the majority of cases. This is not a new finding; between 1997 and 2002, 18% of articles in five major medical journals did not report informed consent or REC approval [22]. Similarly, a study of 1189 articles in six anaesthesiology journals found 66% reported informed consent and and 71% REC approval respectively and considerable variation was identified between journals [35]. More recent studies also show variation across journals with respect to reporting practices. Murphy and colleagues’ study in 2015 found that 42.9% of articles published in three leading otolaryngology journals did not specify whether informed consent had been provided by research participants [36]. Wu et al.’s study of 2041 articles in five nursing journals published in 2019 found that 87.5% and 93.7% recorded informed consent and REC approval respectively [37]. However, a similar study of 40,278 articles in 12 Chinese nursing journals in 2017 found 27.4% and 51.8% reporting of informed consent and REC approval respectively [38].

Patient autonomy is preserved in clinical research by ensuring that the patient is fully informed before signing a consent form and undergoing any study-specific-procedures.

This review cannot identify whether the consent reported was, in fact, fully informed. Regulatory authorities and monitoring by trial sponsors, where applicable, also have an important role to play in ensuring ethical standards are maintained. There are multiple reasons why fully informed consent is challenging to achieve in a pandemic setting, and the acceptability of other models of consent (deferred, third party or waived) in medical emergency settings has been reviewed by Gobat previously [39]. Participants had mixed opinions about prospective consent in emergency settings, and third party consent was acceptable to many [39]. Studies involving waived consent in the Gobat review were subject to legislative safeguards; in Ireland this safeguard is provided by the Health Research Consent Declaration Committee [40]. The Gobat review was conducted to identify the acceptability of different consent models in a pandemic setting but very few of the papers reviewed were directly relevant—highlighting that research into methodology and medical ethics is lacking in a pandemic setting.

Requirements with regard to reporting of procedures in publications is becoming increasingly standardized, for example the use of the PRISMA [19] or Critical Appraisal Skills Program (CASP) Checklists [41]. There is a reference to informed consent in the Case Report Guidelines (CARE) checklist [42] but no ethical safeguards are included in the most commonly used checklists for the reporting of Observational studies (Strengthening the Reporting of Observational Studies in Epidemiology—STROBE [43]) or randomised trials, (Consolidated Standards of Reporting Trials—CONSORT [44]). Standardized reporting of ethical safeguards including REC approval, informed consent and a statement regarding participant confidentiality should also be a priority, perhaps by updating already established checklists to include the ethical safeguards which are generally applicable to these kinds of clinical research.

### Strengths and limitations

This review incorporated the biggest database of medical and biomedical literature and this permitted a broad assessment of how well COVID-19 medical research ethical standards reporting at two points in the pandemic. However, streamlining the systematic review process into a rapid review, e.g., only a single researcher performing each data extraction, may have introduced some level of bias [45]. Similarly, there are limitations associated with only reviewing articles published in the English language and had the full free text available. It is also important to note that the Declaration of Helsinki and GCP are not systematically enforced by law, so this may explain why some of the articles identified in this rapid review do not report compliance with these ethical guidelines.

## Conclusions

While the majority of journal’s editorial policies purported to support the ICMJE or COPE guidelines, or specifically mentioned the key four ethical parameters, many COVID-19 clinical research publications identified in this rapid review lacked documentation of these important safeguards for research participants. However, there are indicators that reporting of these safeguards is improving as the pandemic is progressing. In order to promote public trust, ethical declarations should consistently be included in publications of clinical research.

## Supplementary Information

**Additional file 1**. PRISMA 2009 Checklist.

**Additional file 2**. PRISMA-S Checklist.

**Additional file 3**. Data Extraction Form Template.

**Additional file 4**. Extracted Data - April search.

**Additional file 5**. Extracted Data - November search.

**Additional file 6**. Rapid Review protocol.

## Data Availability

All data generated or analysed during this study are included in this published article and its supplementary information files.

## Abbreviations

CASP: Critical Appraisal Skills Program
CONSORT: Consolidated Standards of Reporting Trials
COVID-19: Coronavirus disease 2019
CTIMP: Clinical Trial of an Investigational Medicinal Product
EU: European Union
GCP: Good Clinical Practice
GDPR: General Data Protection Regulation
ICH: International Conference on Harmonisation of Technical Requirements for the Registration of Pharmaceuticals for Human Use
ICMJE: International Committee of Medical Journal Editors
MEDLINE: Medical Literature Analysis and Retrieval System Online
PRISMA: Preferred Reporting Items for Systematic Reviews and Meta-Analyses
REC: Research Ethics Committee
STROBE: Strengthening the Reporting of Observational Studies in Epidemiology
