# Supplementary material for: Adherence with reporting of ethical standards in COVID-19 human studies: a rapid review
Source: BMC Med Ethics. 2021 Jun 28;22:80. doi: 10.1186/s12910-021-00649-9 (PMC8237766; doi:10.1186/s12910-021-00649-9)
Supplement: Supplementary file 6 — Additional file 6. Rapid Review protocol. [file 12910_2021_649_MOESM6_ESM.pdf]

Check for updates

STUDY PROTOCOL

REVISED Ethical Considerations for safeguarding human participants in pandemic research: a rapid review protocol [version 2; peer review: 2 approved]

Previously titled: Protocol for systematic review of ethical declarations made in clinical publications concerning COVID-19

Lydia O'Sullivan[1-3], Ronan P. Killeen[4], Peter Doran[1,3], Rachel K. Crowley[1,4]

[1]UCD School of Medicine, University College Dublin, Dublin 4, Ireland
[2]HRB Trials Methodology Research Network, Áras Moyola, National University of Ireland, Galway, Ireland
[3]UCD School of Nursing, Midwifery and Health Systems, University College Dublin, Dublin, Dublin 4, Ireland
[4]Research Ethics Committee, St Vincent's University Hospital, Dublin 4, Ireland

v2 **First published:** 13 May 2020, **3**:22
https://doi.org/10.12688/hrbopenres.13053.1

Latest published: 20 Jul 2020, **3**:22
https://doi.org/10.12688/hrbopenres.13053.2

Abstract
COVID-19 is a respiratory disease caused by a coronavirus, designated SARS-CoV-2, which is responsible for a global pandemic in 2020. Public interest in this disease has led to the publication of thousands of articles in the medical literature in a very short timeframe. It is imperative that medical research into COVID-19 is conducted quickly and safely, and that due reference is given to the ethical considerations enshrined in the ICH GCP guidelines, according to the Declaration of Helsinki.
In order to review the reporting of ethical considerations in these papers, we hereby propose a protocol for a systematic review of COVID-19 papers up to April 14 th 2020. The search criteria proposed for the review are based upon what would be a reasonable search conducted by a lay member of the public with access to PubMed.gov.
Institutional Research Ethics Committees (RECs) face significant challenges in providing thorough and timely ethical review during the COVID-19 pandemic. It is proposed to publish the findings of this rapid review along with a summary of an institutional REC response to the challenges of reviewing and approving clinical research proposals in the time of a pandemic.

Keywords
COVID-19, pandemic, research ethics, clinical trials, case studies

Open Peer Review

Reviewer Status ✓ ✓

	Invited Reviewers	
	1	2
version 2 (revision) 20 Jul 2020	✓ report	✓ report
version 1 13 May 2020	? report	? report

1. **Linda Biesty**, National University of Ireland Galway, Galway, Ireland

2. **Bethany Spielman**, Southern Illinois University, Carbondale, USA

Any reports and responses or comments on the article can be found at the end of the article.

This article is included in the Coronavirus (COVID-19) collection.

This article is included in the HRB-TMRN collection.

Corresponding author: Lydia O'Sullivan (lydia.osullivan@ucdconnect.ie)

Author roles: O'Sullivan L: Investigation, Writing – Review & Editing; **Killeen RP**: Methodology, Writing – Review & Editing; **Doran P**: Funding Acquisition, Methodology, Writing – Review & Editing; **Crowley RK**: Conceptualization, Methodology, Visualization, Writing – Original Draft Preparation

Competing interests: No competing interests were disclosed.

Grant information: This study was funded by the Health Research Board, Ireland (grant numbers HRB-TMRN-2017-1, HRB-CRCI-OR-2020-6).
The funders had no role in study design, data collection and analysis, decision to publish, or preparation of the manuscript.

Copyright: © 2020 O'Sullivan L *et al*. This is an open access article distributed under the terms of the Creative Commons Attribution License, which permits unrestricted use, distribution, and reproduction in any medium, provided the original work is properly cited.

How to cite this article: O'Sullivan L, Killeen RP, Doran P and Crowley RK. **Ethical Considerations for safeguarding human participants in pandemic research: a rapid review protocol [version 2; peer review: 2 approved]** HRB Open Research 2020, **3**:22 https://doi.org/10.12688/hrbopenres.13053.2

First published: 13 May 2020, **3**:22 https://doi.org/10.12688/hrbopenres.13053.1

REVISED **Amendments from Version 1**

Protocol V2 includes suggestions from the reviewers. The Title was amended to reflect that the review more accurately fits a Rapid Review methodology. Some sentences were added in or amended to clarify that a description of the Standard Operating Procedures of an Irish Research Ethics Committee pre-COVID 19 will be included in the publication of this Rapid Review, along with how the Committee adapted their procedures to ensure timely and through ethical review during the pandemic. The details of the search strategy and outcome were clarified. Some references were added or amended per the reviewer's advice. Clarification was also provided regarding the rationale for the contributions of the lay persons to this review.

Any further responses from the reviewers can be found at the end of the article

Introduction

In December 2019 the first reports of a novel coronavirus, now designated SARS-CoV-2, emerged from China[1]. Since then SARS-CoV-2 has spread across the globe and is the agent responsible for a viral pneumonitis called COVID-19[2]. Its virulence, the rapidity of its spread and the lack of either a vaccine or a treatment have led to the adoption of unprecedented public health measures worldwide[3]; and to high levels of interest and anxiety in the general population[4].

The international medical and scientific research community has responded to the COVID-19 pandemic by generating a huge amount of literature to which the general public has access[5]. The COVID-19 related publications range from commentary and case reports to epidemiological studies and clinical trials. It is the policy of many journals during the pandemic to offer rapid review in order to make reports and research on COVID-19 available quickly[6]. However, London and Kimmelman have expressed concern that scientific and methodological research standards will fall in the urgency to produce results[7]. Similarly, the authors have observed considerable variability in the reported ethical review of the studies published and seek to conduct a systematic review of this question – how well are ethical disclosures ethical disclosures reported in the COVID-19 literature? Spector-Bagdady et al pointed out that clinicians and medical centres are faced with difficult decisions regarding which trials to participate in and are faced with challenges with implementing both an ethical and efficient recruitment method for COVID-19 trials[8]. Tansey and colleagues also highlighted the importance of having a framework in place for ethical review in an emergency situations[9]. Along with the results of this Rapid Review, a description will be provided of the response of an Irish Ethics Committee to the challenges presented by the COVID-19 pandemic. The Standard Operating Procedures of this Ethics Committee will be presented, along with how these were adapted to ensure thorough and timely ethical review during the COVID-19 pandemic.

Protocol

Protocol design and registration

The protocol is designed in line with the PRISMA-P checklist for systematic review[10,11]. The protocol is not eligible for registration on the PROSPERO database, as the question of interest relates to reporting and methodology rather than to a clinical outcome for participants.

The ethical standards of interest described by Yank and Rennie[12] will be used. These include an assessment of the standards agreed by the International Committee of Medical Journal Editors[13] following the Declaration of Helsinki[14]; the report of consent obtained for the study and the report of ethical review of the study.

The search parameters are: Pubmed search of the term "COVID-19" with the filters of Free full text and English language publication – the rationale for this search is that it would be reasonable for a lay member of the public to conduct such a search. No start date is applied and the finish date of the search is April 14th 2020.

Search record and outcome management

The bibliography of the search will be saved and the assessors will use an Excel spreadsheet to record excluded and included studies, using the reference number from the search bibliography. Papers may be included if they are defined by an assessor as a case report, case series, observational study or clinical trial. Papers will be excluded if they are classified as a commentary, editorial, review, guideline, non-clinical report, cell-based study, animal study, epidemiologic study or report of a mathematical model. Two reviewers will conduct the initial assessment (half of the papers each, identified by the initial search). The reviewers will confer where there is any uncertainty.

Two independent reviewers will conduct a bias assessment. Each independent reviewer will assess 10 studies each for each initial assessor, identified randomly from the search bibliography (20 papers in total by each independent assessor). A threshold of 20% discrepancy between initial and independent assessors has been set to determine whether a further methodological revision is required. A further five records each will be reviewed following this initial method assessment.

For case studies and case series, the assessor will record: the first three authors; journal; study title; whether written consent reported; if not written consent whether oral consent reported or whether not available (i.e. patient deceased); whether written consent to publish provided to journal; the wording of consent; the nature of identifiers reported (such as location, occupation etc); whether the article included a statement of compliance with International Conference on Harmonisation (ICH) good clinical practice (GCP)/Declaration of Helsinki standards.

For observational studies and clinical trials, the assessor will record: the first three authors; journal; study title; whether written consent reported; the wording of consent; if not written consent whether oral consent reported; review by research ethics committee; whether that committee is identifiable on line; whether the article included a statement of compliance with ICH GCP/Declaration of Helsinki standards and in the case of a clinical trial, whether the trial was registered.

Analysis

The outcome is the proportion of papers where informed consent to publish (in case studies and series) and for inclusion in a clinical research study as well as publication (observational studies and clinical trials) was recorded. An assessment of the role of ethics committees will be included; specifically, whether ethics committee approval was sought and whether requirement for consent was waived by said committee and whether there is a geographical or publication pattern associated with the reported role of the ethics committee.

This is a methodologic review; therefore, bias in interpretation of results is not relevant to this study. The proposed assessment of the ethics reporting standard categorized by journal may identify a publication bias.

A quantitative summary of numbers of studies identified, and their classification by assessor, will be provided. A further summary of consent reported and the nature of that consent will be prepared. The proportion of studies reporting ethical review and including a statement of compliance with ethical guidelines for conduct of clinical research studies (observational study or clinical trial), will be included. A qualitative description of the measures to preserve anonymity of the participants will be made.

Ethical review and lay opinion

This study will not be clinical in nature and does not meet the requirement for a research ethics committee review. In view of the nature of the study and the impact of ethical conduct of research on public confidence in research, it is proposed to share the final draft of the study manuscript with two lay volunteers for a lay perspective on the findings prior to submission for publication. The protocol will be made available for the public on the UCD COVID-19 website.

Future dissemination and availability of data

The search bibliography and finalized Excel spreadsheets will be included as supplementary documents with the final publication. As a publication relating to COVID-19, it is anticipated that this will be open access. Should this not be the case the authors will make these files available on request to the corresponding author.

Conclusions

The purpose of conducting this review is to describe the existing publication status for ethical declarations in COVID-19 clinical research and discuss the potential impact on public trust in clinical research. It is proposed to include the findings with a description of how n Irish Research Ethics Committee conducted ethical assessment both expeditiously and safely in a time of pandemic; in order to maintain ethical standards, maximize public access to participation in clinical trials and research, and maintain public confidence in future research studies and their findings.

Data availability

Underlying data

No data are associated with this article.

Reporting guidelines

Figshare: PRISMA-P checklist for 'Protocol for systematic review of ethical declarations made in clinical publications concerning COVID-19'. https://doi.org/10.6084/m9.figshare.12249224.v1[11].

The completed PRISMA-P checklist is available under the terms of the Creative Commons Attribution 4.0 International license (CC-BY 4.0).

Author contributions

All authors contributed to the protocol design. LOS and RC drafted the manuscript which was approved by all authors. Funding was acquired by PD.

References

1. Zheng J: **SARS-CoV-2: an Emerging Coronavirus that Causes a Global Threat.** *Int J Biol Sci.* 2020; **16**(10): 1678–85.
PubMed Abstract | Publisher Full Text | Free Full Text
2. Jiang F, Deng L, Zhang L, *et al.*: **Review of the Clinical Characteristics of Coronavirus Disease 2019 (COVID-19).** *J Gen Intern Med.* 2020.
PubMed Abstract | Publisher Full Text
3. WHO: **Coronavirus disease 2019 (COVID-19) situation report—56.** 2020.
Reference Source
4. Wang C, Pan R, Wan X, *et al.*: **Immediate Psychological Responses and Associated Factors during the Initial Stage of the 2019 Coronavirus Disease (COVID-19) Epidemic among the General Population in China.** *Int J Environ Res Public Health.* 2020; **17**(5): pii: E1729.
PubMed Abstract | Publisher Full Text | Free Full Text
5. **Public Health Emergency COVID-19 Initiative: PubMed.** 2020. Accessed 15th July 2020.
Reference Source
6. Wellcome: **Sharing research data and findings relevant to the novel coronavirus (COVID-19) outbreak.** 2020.
Reference Source
7. London AJ, Kimmelman J: **Against pandemic research exceptionalism.** *Science.* 2020; **368**(6490): 476–477.
PubMed Abstract | Publisher Full Text
8. Spector-Bagdady K, Higgins PDR, Lok AS: **COVID-19 Clinical Trial Oversight at a Major Academic Medical Center: Approach of the Michigan Medicine COVID-19 Clinical Trial Committees.** *Clin Infect Dis.* 2020; ciaa560.
PubMed Abstract | Publisher Full Text | Free Full Text
9. Tansey CM, Herridge MS, Heslegrave RJ, *et al.*: **A framework for research ethics review during public emergencies.** *CMAJ.* 2010; **182**(14): 1533–7.
PubMed Abstract | Publisher Full Text | Free Full Text
10. Moher D, Shamseer L, Clarke M, *et al.*: **Preferred reporting items for systematic review and meta-analysis protocols (PRISMA-P) 2015 statement.** *Syst Rev.* 2015; **4**(1): 1.
PubMed Abstract | Publisher Full Text | Free Full Text
11. O'Sullivan L, Kileen R, Doran P, *et al.*: **PRISMA-P-checklist - COVID-19 Research

Ethics Review. *figshare*. Figure. 2020.
http://www.doi.org/10.6084/m9.figshare.12249224.v1

12. Yank V, Rennie D: **Reporting of informed consent and ethics committee approval in clinical trials.** *JAMA.* 2002; **287**(21): 2835–38.
PubMed Abstract | Publisher Full Text

13. Editors ICoMJ: **Recommendations for the conduct, reporting, editing, and publication of scholarly work in medical journals.** accessed 10th January 2020.
Reference Source

14. Declaration of Helsinki: **Helsinki, Finland: World Medical Association.** 1964.
Reference Source

Open Peer Review

Current Peer Review Status: ✔ ✔

Version 2

Reviewer Report 12 August 2020

https://doi.org/10.21956/hrbopenres.14214.r27696

© 2020 Spielman B. This is an open access peer review report distributed under the terms of the Creative Commons Attribution License, which permits unrestricted use, distribution, and reproduction in any medium, provided the original work is properly cited.

Bethany Spielman
Southern Illinois University, Carbondale, IL, USA

I am satisfied with the changes; it can be indexed

Competing Interests: No competing interests were disclosed.

Reviewer Expertise: Bioethics and law of human subjects research.

I confirm that I have read this submission and believe that I have an appropriate level of expertise to confirm that it is of an acceptable scientific standard.

Reviewer Report 04 August 2020

https://doi.org/10.21956/hrbopenres.14214.r27697

© 2020 Biesty L. This is an open access peer review report distributed under the terms of the Creative Commons Attribution License, which permits unrestricted use, distribution, and reproduction in any medium, provided the original work is properly cited.

Linda Biesty
School of Nursing and Midwifery, National University of Ireland Galway, Galway, Ireland

Thank you to the team for updating the protocol and providing Version 2. I wish you all the very best with this review.

Competing Interests: No competing interests were disclosed.

Reviewer Expertise: Qualitative research, trial methodology, evidence synthesis (qualitative and quantitative).

I confirm that I have read this submission and believe that I have an appropriate level of expertise to confirm that it is of an acceptable scientific standard.

Version 1

Reviewer Report 10 June 2020

https://doi.org/10.21956/hrbopenres.14149.r27481

© **2020 Spielman B.** This is an open access peer review report distributed under the terms of the Creative Commons Attribution License, which permits unrestricted use, distribution, and reproduction in any medium, provided the original work is properly cited.

?

Bethany Spielman
Southern Illinois University, Carbondale, IL, USA

This can be a useful study. I look forward to reading it upon completion. The study could be improved by addressing several issues described below:

Introduction
The following literature should be engaged/cited:
London and Kimmelman (2020)[1]; Spector-Bagdady *et al.* (2020)[2]; Tansey *et al*. (2010)[3].

Abstract
The abstract states that the study will publish a summary of the ethics committee responses to challenges of reviewing and approving protocols during a pandemic, but the protocol does not have a way of identifying what those challenges are or what committee responses to the pandemic situation have been. Some committees may always have been lax; the pandemic situation may not be more lax than their baseline.

Protocol
Protocol Design and Registration:
Please consider adopting completely Yank's categories for describing informed consent. Or, alternatively, explain: a) why you are not doing so and b) how readers should compare Yank's outcomes to yours. Comparison could be an important step in understanding pandemic constraints/responses if you make a greater effort to make your study comparable to Yank's.

"Search Record and Outcome Management"
For case studies and case series, the "wording of consent" will be recorded. It will not be recorded for observational studies and clinical trials. Why not?

Conclusions
One of the stated purposes of the study is to describe the potential impact on public trust in clinical research. What are you going to ask lay people at the end of the study that will help determine that impact? Do you have enough lay people to say much? Is this really a second phase

of the study?

The conclusion states that a "description of how ethical assessment can be expedited safely in a time of pandemic…." will be included. The proposed study will not support any conclusions about whether the way things were done in the COVID 19 studies was expeditious or safe, or whether public access to participation was maximized. It will support conclusions about whether certain ethical standards were maintained (particularly if Yank's outcomes are used as a touchpoint); and it may (if the lay role is made more rigorous) support conclusions about public confidence.

References

1. London AJ, Kimmelman J: Against pandemic research exceptionalism.*Science*. 2020; **368** (6490): 476-477 PubMed Abstract | Publisher Full Text
2. Spector-Bagdady K, Higgins PDR, Lok AS: COVID-19 Clinical Trial Oversight at a Major Academic Medical Center: Approach of the Michigan Medicine COVID-19 Clinical Trial Committees.*Clin Infect Dis*. 2020. PubMed Abstract | Publisher Full Text
3. Tansey CM, Herridge MS, Heslegrave RJ, Lavery JV: A framework for research ethics review during public emergencies.*CMAJ*. 2010; **182** (14): 1533-7 PubMed Abstract | Publisher Full Text

Is the rationale for, and objectives of, the study clearly described?

Yes

Is the study design appropriate for the research question?

Yes

Are sufficient details of the methods provided to allow replication by others?

Yes

Are the datasets clearly presented in a useable and accessible format?

Not applicable

Competing Interests: No competing interests were disclosed.

Reviewer Expertise: Bioethics and law of human subjects research.

I confirm that I have read this submission and believe that I have an appropriate level of expertise to confirm that it is of an acceptable scientific standard, however I have significant reservations, as outlined above.

Author Response 15 Jul 2020

Rachel Crowley, University College Dublin, Dublin 4, Ireland

Thank you Bethany for your time in reviewing our protocol. We appreciate all of your comments and suggested changes. We have addressed each of the points you have raised and amended the protocol accordingly.

Thank you for highlighting these publications. We have added them to the Introduction

section.

The final publication will include a description of the Standard Operating Procedures of a REC which will demonstrate the rigour of their review pre-COVID 19. The publication will also identify how they adapted their operational procedures to ensure timely and thorough ethical review during the COVID-19 pandemic. The Introduction and Conclusion have been amended to explain this.

The outcomes described by Yank and Rennie will be used. The wording in the 'Protocol design and registration' section has been amended to clarify this.

The 'wording of consent' will be recorded for observational and clinical trials, so this sentence was amended accordingly.

The purpose of the review is to determine to what extent COVID-19 publications report ethical declarations, given that the lack of these declarations may damage public trust in research. We have amended the wording of this section slightly to clarify this. The purpose of requesting two lay persons to review the manuscript was to incorporate their opinions, as well as the viewpoints of clinical researchers on the findings of this review. A larger exploration of lay opinions on COVID-19 research would indeed be valuable and interesting but it is unfortunately not within the scope of this research.

This wording referred to the description of the Standard Operating Procedures of an Irish Research Ethics Committee in pre-COVID 19 (please see amended text in the Introduction), which will be included in the publication. Along with this description, an account will be given of how this Ethics Committee adapted their practices to ensure timely and thorough ethical review despite the challenges presented by the COVID-19 pandemic. We have amended the wording of the Conclusion to clarify this.

Many thanks again Bethany.

Competing Interests: No competing interests were disclosed.

Reviewer Report 26 May 2020

https://doi.org/10.21956/hrbopenres.14149.r27403

© 2020 Biesty L. This is an open access peer review report distributed under the terms of the Creative Commons Attribution License, which permits unrestricted use, distribution, and reproduction in any medium, provided the original work is properly cited.

?

Linda Biesty
School of Nursing and Midwifery, National University of Ireland Galway, Galway, Ireland

I want to start by congratulating the team on their proposal. This is an important study and I was

delighted to have the opportunity to offer some thoughts and comments.

Some points of feedback and comments:

The Title of the Study: I suggest that the title needs to be reviewed. At present it does not indicate clearly to a reader that "ethical declarations" relate only to the reporting of informed consent and ethics committee approval. The teams definition of 'Clinical publications' also needs to be explicit in the title and the 'type' of studies to be included needs to remain consistent throughout the protocol.

Abstract: The last point in the abstract is not clear. What is meant by publishing the findings of this review "with" a summary of the IREC response to the challenges of reviewing and approving clinical research proposals in the time of pandemic? If the review authors intend to use the IREC response as a step in their interrogation of the findings this needs to be clear.

Introduction: The review authors draw attention to the Torres-Salinas paper presenting "a global vision of the daily growth of scientific production on Covid-19 in various databases". I was able to access only an English translation of the abstract of this paper, therefore, how it links to the amount of literature the general public can access, remains unclear.

The question identified at the end of this section (nature of ethical disclosure) is broader than the focus of the review as documented in other places (reporting of informed consent and ethics committee approval) this needs to be realigned.

Protocol: Some rational needs to be provided as to why the review authors state that it is "reasonable for a lay member of the public to conduct such a search". Is there evidence to suggest that the use of Pubmed (and Pubmed only) is appropriate? Have the different variations of the terms of COVID-19 been considered and how will the search account for this? Will this review include pre-prints and what considerations have been given to the time frame between the availability of a publication and its indexing in Pubmed?

Search record and outcome management: Where does qualitative research fit within the inclusion / exclusion criteria?

Analysis: It is not clear what is meant by "a qualitative assessment", this needs to be clarified. The analysis section notes that a qualitative description of the measures to preserve anonymity – however no reference is made to extracting data relating specifically to anonymity.

Ethical review and lay opinion: Including a lay perspective in the review is welcome, however, the role of the 2 lay volunteers is not clear. Will the volunteers be asked offer their opinions in relation to the accuracy of the review, the rigour of your methods or the readability of the paper?

Conclusions: The claims noted in this section need to be revisited. The purpose of this review noted on line 2 of this section denotes that the review will focus on "ethical standards in COVID-19 clinical trials" – this is not in keeping with the rest of the protocol. Nor does the protocol indicate how this review can highlight issues in relation to public trust. It is not clear how the review will provide a description of how ethical assessment can be expedited safely in a time of pandemic. Nor is it clear how the findings of this review will support the assumption that reporting ethical

approval equates to maintaining ethical standards. The link between reporting the findings of this review and the " ... public's confidence in future research and their findings" also needs to be reconsidered.

Is the rationale for, and objectives of, the study clearly described?
Partly

Is the study design appropriate for the research question?
Yes

Are sufficient details of the methods provided to allow replication by others?
Partly

Are the datasets clearly presented in a useable and accessible format?
Not applicable

Competing Interests: No competing interests were disclosed.

Reviewer Expertise: Qualitative research, trial methodology, evidence synthesis (qualitative and quantitative).

I confirm that I have read this submission and believe that I have an appropriate level of expertise to confirm that it is of an acceptable scientific standard, however I have significant reservations, as outlined above.

Author Response 15 Jul 2020

Rachel Crowley, University College Dublin, Dublin 4, Ireland

Thank you Linda for your time in reviewing our protocol. We appreciate all of your comments and suggested changes. We have addressed each of the points you have raised and amended the protocol accordingly.

Title was amended to reflect the reviewer's comment and also because the authors feel that the review more accurately fits the Rapid Review methodology. Title now reads: *Ethical Considerations for safeguarding human participants in pandemic research: a rapid review protocol*

The wording of 'clinical publications' has been amended throughout to clarify and ensure consistency.

A description of the response of an Irish Ethics Committee to the challenges presented by the COVID-19 pandemic will be provided in the Discussion section of the Rapid Review to complement the Rapid Review findings. Operational and logistical challenges in providing thorough and timely ethical review during the COVID-19 pandemic will be discussed. The Introduction and Conclusion were amended to clarify this.

The Torres-Sallinas reference was removed and a reference to an announcement on the PubMed website that over 50 publishers have made COVID-19 publications accessible was added.

The sentence at the end of the Introduction (nature of ethical disclosure) was amended to clarify scope of this review.

PubMed facilitated over 50 publishers in providing access to full-text publications relating to COVID-19: https://www.ncbi.nlm.nih.gov/pmc/about/covid-19/ so the rationale was that this platform would make these publications accessible to members of the public. A reference to this webpage has been added to the protocol (see above point).

The purpose of this rapid review was to assess how well a representative sample of human research on COVID-19 adhered to ethical guidelines such as informed consent, REC review etc, rather than to perform an exhaustive search of all COVID-19 publications. For this reason, a single but comprehensive database was selected, which provides full-text access to lay people. Due to the fact that pre-prints have not undergone peer review, they will not be included in this review, as the intent is to assess peer reviewed articles. However, 'Epub ahead of print' articles will be included, as they have undergone peer review.

A search for 'COVID-19' in PubMed yields the following terms: "COVID-19"[All Fields] OR "severe acute respiratory syndrome coronavirus 2"[All Fields] OR "severe acute respiratory syndrome coronavirus 2"[All Fields] OR "2019-nCoV"[All Fields] OR "SARS-CoV-2"[All Fields] OR (("Wuhan"[All Fields] AND ("coronavirus"[MeSH Terms] OR "coronavirus"[All Fields])) AND 2020[All Fields]). It was therefore felt that that the term 'COVID-19' would provide us with a broadly representative sample of COVID-19 research publications.

No filters will be applied other than 'Free Full Text' and 'English Language' so qualitative studies will be included. A initial search indicated that the qualitative COVID-19 studies were observational in nature, so they will be included in this category.

A review of whether publications record if REC approval was sought and whether the requirement for consent was waived by the REC will be conducted. If the information is available, a review will be conducted of the geographical or publication patterns also. We have removed the word 'qualitative'.

Under 'Search Record and Outcome Management', one of the items that will be recorded is 'nature of identifiers'. We have added '(such as location, occupation etc)' to clarify the meaning of this.

The purpose of requesting two lay persons to review the manuscript was to incorporate their opinions, as well as the viewpoints of clinical researchers on the findings of this review. We have amended this sentence to clarify.

The final publication will include a description of the Standard Operating Procedures of a REC which will demonstrate the rigour of their review pre-COVID 19. The publication will also identify how they adapted their operational procedures to ensure timely and thorough

ethical review during the COVID-19 pandemic. The Conclusion has been re-worded to address this comment.

Many thanks again Linda.

Competing Interests: No competing interests were disclosed.
